# SMARCB1 Promotes Ubiquitination and Degradation of NR4A3 via Direct Interaction Driven by ROS in Vascular Endothelial Cell Injury

**DOI:** 10.1155/2020/2048210

**Published:** 2020-10-23

**Authors:** Bingzheng Lu, Zhu Zhu, Longxiang Sheng, Yuan Li, Yang Yang, Yupin Chen, Dongdong Xue, Yuwei Zhou, Wei Cai, Chen Chen, Cailv Wei, Dong Xu, Min Yan, Suizhen Lin, Guangmei Yan, Wei Yin

**Affiliations:** ^1^Department of Pharmacology, Zhongshan School of Medicine, Sun Yat-sen University, Guangzhou 510080, China; ^2^Guangzhou Cellprotek Pharmaceutical Co., Ltd., G Building F/4, 3 Lanyue Road, Science City, Guangzhou 510663, China; ^3^State Key Laboratory of Ophthalmology, Zhongshan Ophthalmic Center, Sun Yat-sen University, Guangzhou 510060, China; ^4^Department of Biochemistry, Zhongshan School of Medicine, Sun Yat-sen University, Guangzhou 510080, China; ^5^Department of Pathology, The First Affiliated Hospital of Sun Yat-sen University, Guangzhou 510080, China

## Abstract

Nuclear receptor subfamily 4 group A member 3 (NR4A3) protects the vascular endothelial cell (VEC) against hypoxia stress, whose expression is primarily reported to be governed at a transcriptional level. However, the regulation of NR4A3 in the protein level is largely unknown. Here, we report that NR4A3 protein abundance is decreased immensely in VEC injury induced by reoxygenation after oxygen-glucose deprivation (OGD-R), which is significantly blocked by the administration of the antioxidative steroid TRIOL. Moreover, the notable improvement of NR4A3 and the alleviation of pulmonary endothelial barrier hyperpermeability induced by acute hypobaric hypoxia in cynomolgus monkeys are also observed after TRIOL administration. The overproduction of reactive oxygen species (ROS) decreases NR4A3 protein abundance in VEC under OGD-R condition, which is reversed by TRIOL and N-acetylcysteine (NAC). TRIOL dose-dependently increases the NR4A3 protein level by inhibiting ubiquitination and ubiquitin proteasome system- (UPS-) mediated degradation rather than promoting its transcription. Using yeast two-hybrid screening, we further identify the interaction between NR4A3 and SWI/SNF-related matrix-associated actin-dependent regulator of chromatin subfamily B member 1 (SMARCB1), and the DNA-binding domain of NR4A3 is required for this interaction. Knockdown of SMARCB1 reduces ubiquitination and degradation of NR4A3, suggesting the proubiquitylation effect of this interaction which is enhanced by ROS in VEC injury induced by OGD-R. In summary, our study here for the first time reveals a posttranslational regulation in SMARCB1-mediated NR4A3 protein degradation which is driven by ROS, providing further understanding of the impaired regulation of NR4A3-mediated prosurvival pathways under pathological condition in VEC.

## 1. Introduction

The vascular endothelial cell (VEC) is the core composition of the endothelial barrier, and its injury induced by various pathological stimuli such as ischemia-reperfusion or hypoxia leads to endothelial barrier hyperpermeability [1–3]. In the pathological progress of acute lung injury (ALI), the impairment of the pulmonary endothelial barrier gives rise to the leakage of blood cells and fluids from the circulation into the interstitial space and alveoli, resulting in pulmonary edema and impaired gas exchange followed by the development of hypoxemia and even systemic organ damage [4, 5]. The pulmonary endothelial barrier hyperpermeability mediated by VEC injuries is considered to be an important pathological feature of ALI [1–3]. Nuclear receptor subfamily 4 group A member 3 (NR4A3), also known as neuron-derived orphan receptor-1 (NOR1), acts as a transcriptional activator and is involved in the regulation of physiological and pathological processes such as metabolism, inflammation, cell proliferation, apoptosis, and differentiation [6]. Knockdown of NR4A3 significantly inhibits the transformation of the capillary tube, indicating the vital physiological function of NR4A3 in VEC [7]. Previous research has demonstrated that the upregulation of NR4A3 promotes cell survival under different pathological injuries. For example, the enhanced NR4A3 improves VEC survival under hypoxia condition by upregulating cellular inhibitor of apoptosis 2 (cIAP2) against cell apoptosis [8] and protects neurons from oxidative stress and excitotoxicity induced by glutamate [9].

As an early immediate-response gene, NR4A3 can be induced quickly by various pathological stimuli including hypoxic/ischemic stress, metabolic stress, inflammatory stimuli, and cytokines [10, 11]. Moreover, NR4A3 expression is believed to be governed at the transcriptional level, and hypoxia-inducible factor 1-*α* (HIF1-*α*) and cAMP response element-binding protein (CREB) are identified as the upstream controllers under hypoxia condition [11–13] or growth factor stimulus [14, 15], respectively. The ubiquitin proteasome system (UPS) may participate in the modulation at the protein level for other NR4A family members. Nur77, the first member of the NR4A family (NR4A1), has been reported to be modified by RING-type E3 ubiquitin ligase of tripartite motif 13 (Trim13) and then degraded [16]. However, the possibility and detailed mechanism for UPS to regulate NR4A3 are required to be further identified.

SWI/SNF-related matrix-associated actin-dependent regulator of chromatin subfamily B member 1 (SMARCB1), also known as SNF5 or BAF47, is one of the core subunit proteins of the ATP-dependent SWI/SNF chromatin remodeling complex which plays the key role in transcriptional activation [17]. SMARCB1 has been characterized as a potent tumor suppressor with the involvement in the regulation of cell proliferation, apoptosis, and differentiation in the development and progression of tumors [18, 19]. However, the emerging evidence for the biochemical functions of SMARCB1 raises the possibility that SMARCB1 takes part in the regulation of target expression in a chromatin remodeling-independent manner. It has been reported that SMARCB1 epigenetically regulates gene expression by modulating the histone acetylation via recruiting histone deacetylase (HDAC) to the target promoter [20, 21]. What is more, SMARCB1 is also reported to modulate the activity of transcription factors such as MYC [22, 23], p53 [24], and GLI1 [25] by direct interaction to control downstream target expression. A recent study has further identified the role of the interaction between SMARCB1 and lncRNA at specific promoter regions in modulating transcriptional activity [26]. SMARCB1 is also expressed in VEC [27]; however, little is known about its role in VEC under physiological or pathological conditions.

We previously reported that 5*α*-androst-3*β*, 5*α*, 6*β*-triol (TRIOL), derived from an endogenous neuroactive steroid [28], protects primary cortical neurons from the hypoxia/reoxygenation-induced injury [29] and attenuated the ischemia/reperfusion-induced retinal damages [30]. The protective effect of TRIOL is related to the reduction of the reactive oxygen species (ROS) accumulation induced by hypoxia/reoxygenation [29] and inhibition of TNF-*α*-induced neutrophil adhesion to endothelial cells [31]. A recent study has reported that TRIOL sufficiently attenuated acute hypobaric hypoxia-induced blood-brain barrier (BBB) disruption and vasogenic edema in cynomolgus monkeys [32], strongly suggesting the protective effect of TRIOL on VEC. However, the brain RNA-seq analysis of cynomolgus monkeys did not show the improvement induced by TRIOL on the NR4A mRNA level [32] (Figure S1). Whether the protection of TRIOL on VEC is linked to regulating NR4A3 protein level is required to be further explored.

Here, we report that enhanced NR4A3 protein abundance induced by TRIOL is accompanied by its reduction of VEC injury *in vitro* induced by reoxygenation treatment after oxygen-glucose deprivation (OGD-R) or of the acute lung damage caused by hypobaric hypoxia in monkeys. Moreover, ROS drives ubiquitination and degradation of NR4A3 under OGD-R condition which is alleviated by TRIOL. We further identify the interaction between SMARCB1 and NR4A3 whose DNA-binding domain (DBD) is needed for the interaction. Knockdown of SMARCB1 decreases the ubiquitination and degradation of NR4A3. Furthermore, ROS enhances this interaction under OGD-R condition which is corresponding to its effect on promoting NR4A3 protein reduction and could be abolished by N-acetylcysteine (NAC). Taken together, our study for the first time reveals a posttranslational modulation manner in protein degradation of NR4A3 mediated by SMARCB1, shedding new light on the governing mechanism of NR4A3 expression in VEC during acute lung injury.

## 2. Materials and Methods

### 2.1. Animals and Ethics Statements

All experiments on cynomolgus monkeys were approved by the Ethics Committee of Zhongshan School of Medicine in Sun Yat-sen University and performed according to the standards of the Animal Research: Reporting of In Vivo Experiments (ARRIVE) guidelines [33]. During the experiments, efforts were made to alleviate the sufferings and ensure the welfare of experimental monkeys. The hypobaric hypoxia model of cynomolgus monkeys was employed and detailedly described in our previous report [32]. Briefly, monkeys under normobaric normoxia (NN) condition served as the normal control, while monkeys in the hypobaric hypoxia (HH) group were subjected to a gradiently increased hypobaric hypoxia treatment from 320 m to 7500 m simulated altitude and maintained for 48 hours. Monkeys were euthanized by bloodletting under anesthesia and subjected to subsequent collection and pathological analysis of lung tissues.

### 2.2. Cell Culture, Hypoxia-Related Treatments, and Cytotoxicity Analysis

Human Umbilical Vein Endothelial Cells (HUVEC) (Cellcook, Guangzhou, China) were cultured in modified DMEM (Cellcook, CM2007) supplemented with 10% FBS (Gibco, 10099). Rat vascular endothelial cells (RAOEC) (Jennio-Bio, Guangzhou, China) were cultured in DMEM (Corning, 10-013-CV) supplemented with 10% FBS. The hypoxia-related treatments were carried out using an oxygen-controlled hypoxic working station (HypoxyLab™, Oxford Optronix, UK) with 1% O_2_, 5% CO_2_, 90 ± 1% humidity (RH), and 37.0 ± 0.5°C temperature. Briefly, for OGD, cells were replaced with prehypoxia nutrition-free medium and placed in the hypoxic working station for 4 hours; for OGD-R, cells were subjected to 4-hour OGD treatment and then placed back to normal condition supplied with complete medium for indicated duration. The incubation of drugs, including TRIOL (10 mg/mL, Guangzhou Cellprotek Pharmaceutical Co. Ltd., Guangzhou, China), vehicle (20% HP-*β*-CD), cycloheximide (MCE, HY-12320), chloroquine (MCE, HY-17589), and MG132 (Selleck, S2619), was carried out at the beginning of reoxygenation after OGD with indicated concentration until samples were collected. Lactate dehydrogenase (LDH) in the medium was used to evaluate cytotoxicity at the experimental endpoint with the CytoTox 96 Nonradioactive Cytotoxicity Assay kit (Promega, G1782).

### 2.3. Western Blotting

Cells were washed twice with cold PBS and collected by 1000 g centrifugation at 4°C for 5 min and then lysed using M-PER™ Mammalian Protein Extraction Reagent (Thermo Fisher Scientific, 78501) supplemented with protease inhibitor cocktail (Targetmol, C0001) and phosphatase inhibitor cocktail (Targetmol, C0004). After centrifugation at 12,000 g at 4°C for 10 min, the supernatant was collected. Protein concentrations were determined using a BCA assay kit (Thermo Fisher Scientific, 23225). Then, samples were separated with 10% SDS-PAGE gels and transferred to the PVDF membrane (Roche, 3010040001) followed by blocking with 5% skim milk (Wako, 190-12865). For immunoblotting, the following antibodies were adopted: NR4A3 (Santa Cruz, sc-393902), SMARCB1 (Abcam, ab222519), actin (Arigo, arg62346), GAPDH (Arigo, arg10112), NRF2 (Abcam, ab137550), goat anti-rabbit IgG antibody (HRP) (Arigo, arg65351), and goat anti-mouse IgG antibody (HRP) (Arigo, arg65350). Results were visualized on a ChemiDoc XRS+ system (Bio-Rad Laboratories, USA) with Immobilon Western Chemilum HRP Substrate (Merck Millipore, WBKLS0500).

### 2.4. Immunofluorescence Staining and Hematoxylin-Eosin (HE) Staining

Lung tissues of monkeys were fixed, embedded, and cut into slices routinely, followed by deparaffinization in xylene and hydration in gradient ethanol. Slices were then subjected to antigen retrieval in EDTA solutions (Boster, AR0023). And then, samples were incubated with primary antibodies (NR4A3 and CD31 (Abcam, ab28364)) diluted in an antibody diluent (Dako, S-3022) overnight at 4°C. Then, slices were washed three times with PBS and incubated with fluorescence-conjugated secondary antibodies (Invitrogen, A-21202 and A-31572) at room temperature for 1 hour in the dark. After washing three times and staining with Hoechst 33342 (5 *μ*g/mL, Sigma-Aldrich) at room temperature for 5 min in the dark, slices were mounted and imaged by using a Nikon A1 Spectral Confocal Microscope (Nikon, Japan). All images of different groups were captured with the same settings including pinhole size, image resolution, laser power, and gain value, to ensure the comparability of the fluorescent intensity. Measurement of mean fluorescence intensity (MFI) was conducted with NIS-Elements AR (version 4.60.00, 64-bit) software. HE staining of lung tissues was performed routinely following the manufacturer's instructions of the H&E staining kit (Nanjing Jiancheng Bioengineering Institute, D006). Images for HE staining were captured using an inverted Nikon Eclipse Ti-U microscope (Nikon, Japan).

### 2.5. Determination of C-reactive Protein (CRP), IL-1*β*, and TNF-*α* in Plasma

Blood samples of monkeys in HH and HH+TRIOL groups were collected in anticoagulant tubes before and after the HH treatment. The blood samples then were centrifuged at 3000 rpm for 5 min at room temperature, and the plasma was isolated and stored at -80°C until usage. Determinations of CRP, IL-1*β*, and TNF-*α* were conducted with the following ELISA kits: Monkey C-reactive protein ELISA Kit (mlbio, ML036886-2), Monkey Interleukin-1*β* (IL-1*β*) ELISA Kit (mlbio, ML036883-2), and Monkey Tumor Necrosis Factor Alpha (TNF-*α*) ELISA Kit (mlbio, ML023308-2). All performances were performed routinely following the manufacturer's instructions. The changes of CRP, IL-1*β*, and TNF-*α* in plasma of the HH or HH+TRIOL group are calculated as the content after HH stimulus minus that before HH stimulus, respectively. Statistical significance between the HH and HH+TRIOL groups was determined by using the Mann-Whitney *U* test. A *p* value of <0.05 was considered statistically significant.

### 2.6. ROS Determination and Immunocytochemistry Staining

For ROS determination, cells were stained with CellROX (Invitrogen, C10422) for 30 min according to the manufacturer's instructions under corresponding experimental conditions before the experimental endpoint and then quickly washed and fixed in 4% paraformaldehyde at room temperature for 15 min in the dark, followed by permeabilization in 0.2% Triton/PBS for 15 min. After washing three times with PBS, cells were incubated with a primary antibody diluted in an antibody diluent overnight at 4°C. The following procedures were the same as immunofluorescence staining mentioned above.

### 2.7. Real-Time PCR

Total RNA of cells was extracted using the TRIzol® reagent (Invitrogen, 15596), followed by reverse transcription using oligo (dT) and RevertAid Reverse Transcriptase (Thermo Fisher Scientific, EP0442). Real-time PCR was performed with SuperReal PreMix SYBR Green (Tiangen, FP205) with a 7500 Fast Real-Time PCR System (Applied Biosystems, USA). The relative mRNA expression level was calculated and analyzed by the comparative Ct method (RQ = 2^−*∆∆*Ct^). The sequences of primers are listed as follows.

- *β*-Actin (human): forward: 5′-GATTCCTATGTGGGCGACGA-3′ and reverse: 5′-AGGTCTCAAACATGATCTGGGT-3′
- NR4A3 (human): forward: 5′-AGCGGCGGCATCCTC-3′ and reverse: 5′-CTAAGGGTCCAGGCTCAGG-3′
- SMARCB1 (human): forward: 5′-ACCTAACACTAAGGATCACGGA-3′ and reverse: 5′-CATCCACACCAAAGGGGGAA-3′
- *β*-Actin (rat): forward: 5′-CGCGAGTACAACCTTCTTGC-3′ and reverse: 5′-CGTCATCCATGGCGAACTGG-3′
- NR4A3 (rat): forward: 5′-GGAAACGTGGCGACATCCT-3′ and reverse: 5′-CAGTGGGCTTTGGGTTCTGTG-3′
- SMARCB1 (rat): forward: 5′-AGCTGAACATCCATGTGGGG-3′ and reverse: 5′-GTGGGTTCTCACTGAAGGCA-3′

### 2.8. Yeast Two-Hybrid Screening and Candidate Identification

The Gal4-based yeast two-hybrid screening was carried out according to the manufacturer's protocol (Clontech, cat. PT3247-1). The PCR product of cDNA encoding a C-terminal 308-amino acid fragment (320-627 aa) of NR4A3 (NR4A3-C) was constructed into the Sfi I site of the pGB vector which is a modified pGBKT7 plasmid at multiple cloning sites and used as the bait. The human fetal brain cDNA library purchased from Clontech was subcloned in the pACT2 vector and transfected into Y190 to generate the pool of prey. The positive clones were identified by DNA sequencing and BLASTn search. For further confirmation of interactions in mammalian cells, NR4A3-C was constructed to the pCDEF-flag vector (Shanghai Genomics, Inc.), and candidates were constructed to the pCDEF-HA vector (Shanghai Genomics, Inc.).

### 2.9. Plasmids Construction for Full-Length and Truncated NR4A3

The PCR products encoding full-length and four different truncated NR4A3 fragments were constructed to the pCDEF-flag vector with the following primers. Italic bases indicated sequences for digestion by restriction enzymes.

- Universal forward primer: 5′-*AAAAGGCCAATCCGGCC*ATGCCCTGCGTGCAAGCCCAATATA-3′
- NR4A3-FL (1-627 amino acids) reverse primer: 5′-*AAAAGGCCTCTAAGGCC*TCAGAAAGGCAGGGTATCAAGGAAG-3′
- NR4A3-*Δ*AF2 (1-610 amino acids) reverse primer: 5′-*AAAAGGCCTCTAAGGCCTCA*CAAGTCCTCCAGCTTCAGGTAGAAG-3′
- NR4A3-*Δ*LBD (1-379 amino acids) reverse primer: 5′-*AAAAGGCCTCTAAGGCCTCA*TGGTTTGGAAGGCAGACGACCTCTC-3′
- NR4A3-*Δ*DBD (1-292 amino acids) reverse primer: 5′-*AAAAGGCCTCTAAGGCCTCA*CGTGCCCTCGCCGGATGATGAGCTC-3′
- NR4A3-AF1 (1-124 amino acids) reverse primer: 5′-*AAAAGGCCTCTAAGGCCTCA*CTGGTGGTGGTGGTGATGATGATGG-3′

### 2.10. Immunoprecipitation

Cells were washed twice with cold PBS and collected by 1000 g centrifugation at 4°C for 5 min and then lysed using IP lysis buffer (Beyotime, P0013) supplemented with protease inhibitor cocktail (Targetmol, C0001) and phosphatase inhibitor cocktail (Targetmol, C0004). The supernatant of the cell lysate was collected, and protein concentrations were determined as described above. Then, samples were adjusted to the same concentration and volume and then combined with indicated antibodies and mixed upside down gently and continuously at 4°C overnight. Then, protein A/G immunoprecipitation beads (Bimake, B23202) were added to protein samples and mixed upside down continuously at 4°C for additional 2 hours. The beads of each sample were isolated with a magnetic frame and washed 5 times with IP lysis buffer containing 300 mM NaCl. All samples were eluted with 1X loading buffer (Beyotime, P0015, diluted with lysis buffer) by boiling for 5 min. Samples were subjected to western blot as described above. For immunoprecipitation, the following antibodies were adopted: ubiquitin (Abcam, ab7780), SMARCB1 (Abcam, ab222519), rabbit IgG (Abcam, ab172730).

### 2.11. RNA Interference

The small interfering RNA against SMARCB1 (siSMARCB1) and negative scrambled control (siNC) were purchased from RiboBio (Guangzhou RiboBio Co., Ltd.). RNA interference was conducted with Lipofectamine™ RNAiMAX (Invitrogen, 13778) according to the manufacturer's instructions. Cells that achieved 30~40% confluence were applied to RNA interference treatment. Samples were collected 48 hours after RNA interference treatment and subjected to western blot or real-time PCR as described above. The sequences of siSMARCB1 were listed as follows.

- SMARCB1-human-001: ACGGCGAGTTCTACATGAT
- SMARCB1-human-002: CAGTGTGACCCTGTTAAAA
- SMARCB1-human-003: GAGATTGCCATCCGGAACA
- SMARCB1-rat-001: GAACAGAAGGCCAAGAGAA
- SMARCB1-rat-002: GAATGAGAAGCTAATGACC
- SMARCB1-rat-003: AGACCTATGCCTTCAGTGA

### 2.12. Data Presentation and Statistical Analysis

Data were presented as mean ± SD. Comparative analyses were done by one-way ANOVA, followed by Tukey's multiple comparison tests except for special instructions. A *p* value of <0.05 was considered statistically significant.

## 3. Results

### 3.1. Improved NR4A3 Protein Abundance Is Accompanied by the Protective Effect of TRIOL for VEC under OGD-R/Hypoxia Condition

**Figure 1:**
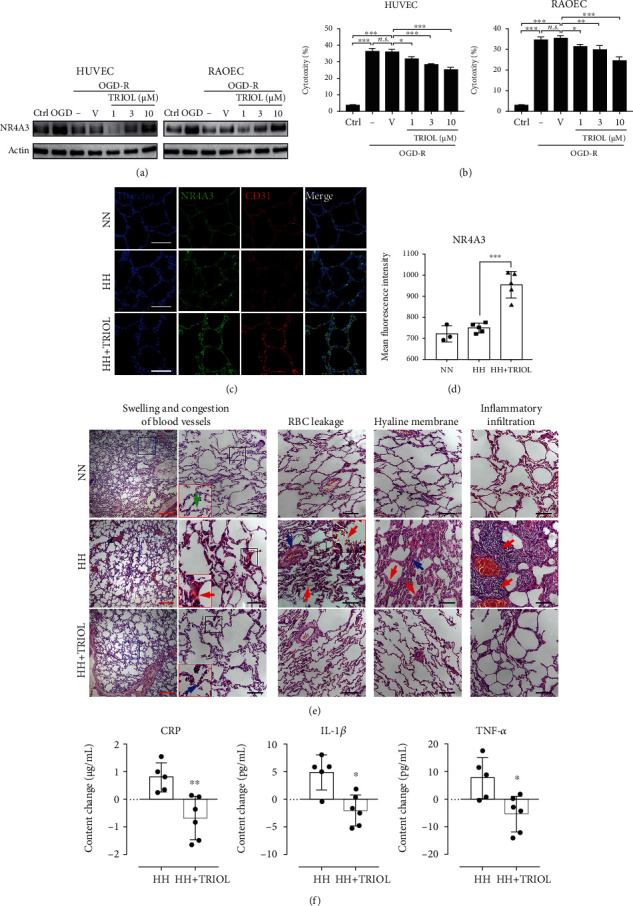
Improved NR4A3 protein abundance is accompanied by the protective effect of TRIOL for VEC under OGD-R/hypoxia condition. (a) Protein expression of NR4A3 in HUVEC (left panel) and RAOEC (right panel) in Ctrl, OGD, and OGD-R condition was examined by western blot. (b) Cytotoxicity to HUVEC (left panel) and RAOEC (right panel) induced by OGD-R was determined by LDH activity in the medium at the experimental endpoint. (c) Representative NR4A3/CD31 double-staining confocal immunofluorescence images of the pulmonary endothelium of the cynomolgus monkeys from normobaric normoxia (NN), hypobaric hypoxia (HH), and hypobaric hypoxia with TRIOL treatment (HH+TRIOL) groups. White scale bar, 100 *μ*m. (d) Quantification of MFI of NR4A3 colocalized with CD31 from (c). The number of individuals of NN, HH, and HH+TRIOL was 3, 5, and 5, respectively. (e) Representative HE staining images of the pulmonary endothelium of monkeys from different groups. Images of typical pathological changes were presented. For panels of swelling and congestion of blood vessels, the views of blue box in left were magnified in right. The views of black box in right were further magnified in the red box. Green, red, and blue arrows indicated the cross sections of capillaries under NN, HH, and HH+TRIOL condition, respectively. For the panels of red blood cell (RBC) leakage, the red arrows indicated leaked RBC in the alveolar cavity and the blue arrow indicated the enlarged perivascular space. The view of black box was further magnified in the red box. For the panels of the hyaline membrane, the red arrows indicated the formation of the hyaline membrane in the surface of the alveolar cavity and the blue arrow indicated fibroplasia in the alveolar septum. For the panels of inflammatory infiltration, the red arrows indicated inflammatory cells infiltrating into the alveolar cavity. Red scale bar, 500 *μ*m; black scale bar, 100 *μ*m. (f) Changes of CRP, IL-1*β*, and TNF-*α* in plasma after acute hypobaric hypoxia treatment in the absence/presence of TRIOL. The content changes are calculated as the content in plasma before HH stimulus minus the content in plasma after HH stimulus. Statistical analysis of (b) and (d) was performed using one-way ANOVA, followed by Tukey's multiple comparison tests; *n.s.*: no significance; ^∗^*p* < 0.05, ^∗∗^*p* < 0.01, and ^∗∗∗^*p* < 0.001. Ctrl: cells cultured in the normal control; OGD: cells subjected to 4-hour OGD treatment; OGD-R: cells subjected to 4-hour OGD treatment followed by 24-hour reoxygenation; V: vehicle; MFI: mean fluorescence intensity.

Given the importance of NR4A3 on regulating cell survival, we firstly explored the NR4A3 protein expression in an injury model of VEC induced by OGD-R. As shown in Figure 1(a), compared with normoxia condition, OGD treatment for 4 hours elevated NR4A3 protein expression which then was reduced by the reoxygenation treatment for 24 hours in HUVEC and RAOEC. The diminishment of NR4A3 induced by OGD-R could be attenuated by TRIOL administration in a dose-dependent manner (Figure 1(a)), and this gradually increased NR4A3 protein abundance by TRIOL is accompanied by the improvement of cell survival (Figure 1(b)). These results indicated that the upregulation of NR4A3 by TRIOL contributed to the protection on VEC *in vitro*, which were further confirmed *in vivo* using an ALI model of nonhuman primate cynomolgus monkeys induced by acute hypobaric hypoxia [33]. As shown in Figures 1(c) and 1(d), NR4A3 was upregulated slightly but not significantly in the pulmonary endothelium under the hypobaric hypoxia condition, while TRIOL observably improved the NR4A3 protein level and reduced the pulmonary endothelium injuries (Figure 1(e)). The pathological examination of lung tissue revealed that the alveolar in the normobaric normoxia (NN) group was polygonal or round, the thin-walled vacuole had a clear boundary, and the cross sections of capillaries could be clearly observed in alveolar septa. Acute hypobaric hypoxia (HH) induced severe tissue swelling, congestion of blood vessels, thickened alveolar septum, locally perivascular edema, erythrocyte deposition, and the formation of hyaline membranes in the alveolar cavity in the HH group (Figure 1(e)). The formation of hyaline membranes is considered to be one of the classic pathological features of diffuse alveolar damage, the hallmark of acute respiratory distress syndrome (ARDS) [34], indicating that permeability of the pulmonary endothelium is increased under acute hypobaric hypoxia condition. Besides, the infiltration of inflammatory cells was also observed (Figure 1(e)). These pathological changes induced by acute hypobaric hypoxia were attenuated by TRIOL administration (HH+TRIOL). To further clarify the protective effect of TRIOL during the ALI, we investigated the contents of several proinflammatory mediators in the plasma including C-reactive protein (CRP), IL-1*β*, and TNF-*α* at the absence/presence of TRIOL under HH condition. Our results showed that acute hypobaric hypoxia induced the evaluation of CRP, IL-1*β*, and TNF-*α* in plasma, which were all significantly suppressed by TRIOL administration (Figure 1(f)), supporting the idea that TRIOL effectively inhibited the systemic inflammation which contributed to the developments of ALI induced by acute hypobaric hypoxia.

Our results here are consistent with the previous observation that the upregulation of NR4A3 protected VEC [8] against hypoxia injury, while TRIOL evaluated NR4A3 and attenuated the injury of VEC induced by OGD-R/hypoxia. How TRIOL regulates the NR4A3 level remains to be further investigated.

### 3.2. ROS Decreases the Protein Level of NR4A3 under OGD-R Condition

**Figure 2:**
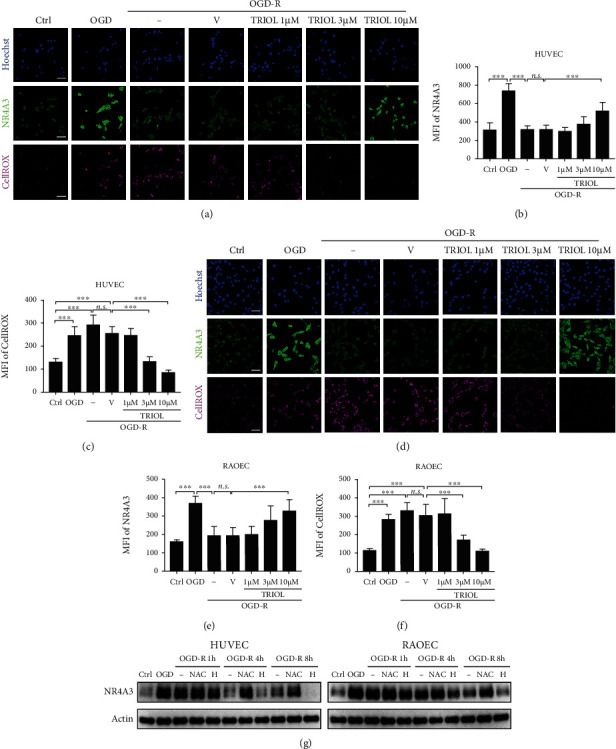
ROS decreases the protein level of NR4A3 under OGD-R condition. (a) Representative NR4A3/ROS double-staining confocal immunofluorescence images of HUVEC in Ctrl, OGD, and OGD-R condition. TRIOL or vehicle was applied at the beginning of reoxygenation. Scale bar, 50 *μ*m. (b) Quantification of MFI of NR4A3 in (a). (c) Quantification of MFI of CellROS in (a). (d) Representative NR4A3/ROS double-staining confocal immunofluorescence images of RAOEC in Ctrl, OGD, and OGD-R condition. TRIOL or vehicle was applied at the beginning of reoxygenation. Scale bar, 50 *μ*m. (e) Quantification of MFI of NR4A3 in (d). (f) Quantification of MFI of CellROS in (d). (g) Protein expression of NR4A3 in HUVEC (left panel) and RAOEC (right panel) subjected to OGD followed by the indicated duration of reoxygenation with/without 4 mM NAC or 400 *μ*M H_2_O_2_ treatment. Statistical analysis of MFI in (b), (c), (e), and (f) was performed using one-way ANOVA, followed by Tukey's multiple comparison tests; *n.s.*: no significance; ^∗∗∗^*p* < 0.001. Ctrl: cells cultured in the normal control; OGD: cells subjected to 4-hour OGD treatment; OGD-R: cells subjected to 4-hour OGD treatment followed by 24-hour reoxygenation; V: vehicle; H: H_2_O_2_; MFI: mean fluorescence intensity.

Numerous researches have demonstrated that overproduction of ROS leads to cell death via activating the proapoptosis pathway, oxidative modification of proteins, nuclear acids, and lipids, and so on, while TRIOL is reported to sufficiently inhibit the neuronal ROS production caused by hypoxia/reoxygenation [29]. Thus, we supposed that the accumulation of ROS is involved in the NR4A3 regulation in VEC damage. As shown in Figures 2(a) and 2(d), overproduction of ROS was significantly induced by OGD followed by reoxygenation treatment. This ROS accumulation was attenuated by TRIOL treatment for 24 h in a dose-dependent manner, while ROS reduction induced by TRIOL was corresponding to gradually increased NR4A3 protein abundance. Furthermore, the diminishment of NR4A3 induced by OGD-R was attenuated by treatment with antioxidant NAC but further enhanced by H_2_O_2_ in a time-dependent manner (Figure 1(g)), confirming that ROS contributes to the regulation of NR4A3 protein abundance. Our finding here indicates that ROS is a critical regulator for NR4A3 in VEC at least under OGD-R condition, and TRIOL and NAC improved the NR4A3 protein level by inhibiting excess ROS production.

### 3.3. NR4A3 Is Degraded via UPS under OGD-R Condition

**Figure 3:**
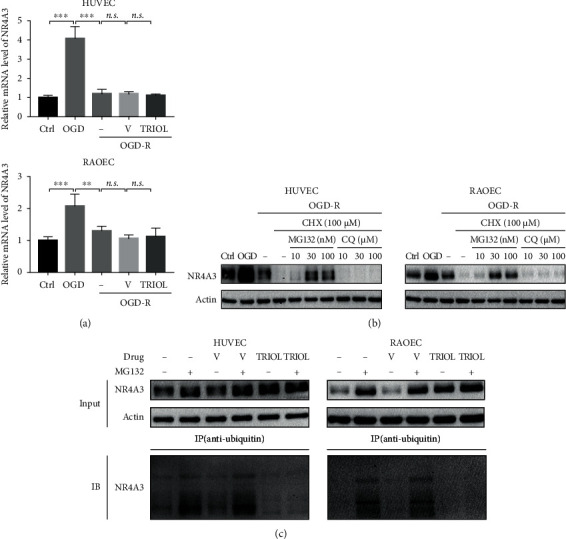
NR4A3 is degraded via UPS under OGD-R condition. (a) Relative mRNA expression of NR4A3 in HUVEC (upper panel) and RAOEC (lower panel) in Ctrl, OGD, and OGD-R condition. 10 *μ*M TRIOL or vehicle was applied at the beginning of reoxygenation. (b) Protein expression of NR4A3 in HUVEC (left panel) and RAOEC (right panel) in Ctrl, OGD, and OGD-R condition. Inhibitors were applied at the beginning of reoxygenation. (c) Determination of ubiquitination of NR4A3 with/without 10 *μ*M TRIOL and/or 100 nM MG132 under OGD-R condition by an antiubiquitin IP assay followed by western blot. Statistical analysis of relative mRNA expression in (a) was performed using one-way ANOVA, followed by Tukey's multiple comparison tests; *n.s.*: no significance; ^∗∗^*p* < 0.01 and ^∗∗∗^*p* < 0.001. Ctrl: cells cultured in the normal control; OGD: cells subjected to 4-hour OGD treatment; OGD-R: cells subjected to 4-hour OGD treatment followed by 24-hour reoxygenation; V: vehicle; CHX: cycloheximide; CQ: chloroquine; input: samples for the co-IP assay; IP: immunoprecipitation; IB: immunoblot.

NR4A3 was primarily reported to be regulated at the transcriptional level which was activated by HIF-*α* under hypoxia condition [5, 14, 15]. Therefore, we further explored the mRNA levels of NR4A3 in VEC after the OGD-R injury. Consistent with the previous reports above, OGD treatment induced the significantly enhanced NR4A3 mRNA in HUVEC and RAOEC which was reversed by OGD-R (Figure 3(a)). Surprisingly, TRIOL showed no obvious effect on mRNA level of NR4A3 under OGD-R condition (Figure 3(a)) or in brain tissue of monkeys (Figure S1) but remarkably enhanced the NR4A3 protein level (Figures 2(a) and 2(d)), strongly suggesting that there is a modulation mechanism of NR4A3 protein level such as the UPS- or lysosome-mediated protein degradation. Thus, we applied cycloheximide (CHX) to inhibit protein synthesis after 4 h OGD treatment and further combined it with MG132 or chloroquine (CQ) to block UPS or lysosomal protein degradation, respectively. The application of CHX resulted in almost complete exhaustion of NR4A3 after OGD-R treatment, which was attenuated dose-dependently by combinational application with MG132 but not with CQ (Figure 3(b)). This result strongly suggested that UPS participated in the regulation of NR4A3 protein abundance in VEC under OGD-R condition. Next, we examined the effect of TRIOL on the ubiquitin modification of NR4A3 in HUVEC and RAOEC after the OGD-R treatment. As shown in Figure 3(c), both proteasome inhibitors MG132 and TRIOL attenuated the reduction of NR4A3 protein caused by OGD-R. However, MG132 treatment but not the combination of MG132 and TRIOL resulted in the accumulation of ubiquitinated NR4A3, indicating that TRIOL attenuated NR4A3 degradation by inhibiting the posttranslational modification of ubiquitination.

### 3.4. SMARCB1 Interacts with NR4A3

**Figure 4:**
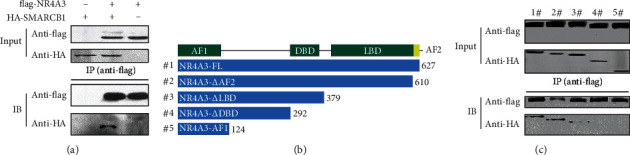
SMARCB1 is identified to be a NR4A3-binding protein. (a) Interaction between NR4A3 and SMARCB1 by the co-IP assay. NR4A3-C (C-terminal 308 amino acids of NR4A3) fused with a flag tag in the N-terminal and SMARCB1 fused with an HA tag in the N-terminal were coexpressed in HEK-293T cells, followed by an antiflag IP assay. Samples for co-IP (input) and collections from co-IP were subjected to western blot. NR4A3-C or SMARCB1 expressed alone served as the negative control. (b) Schematic diagram for the primary structure of full-length and truncated NR4A3 numbered from #1 to #5. Numbers at the end of each fragment corresponded to that of the primary sequence of full-length NR4A3, indicating the length of each fragment. (c) Identification of the interaction between SMARCB1 and full-length or truncated NR4A3, respectively, by the co-IP assay. Full-length or truncated NR4A3 fused with a flag tag in the N-terminal and SMARCB1 fused with an HA tag in the N-terminal were coexpressed in HEK-293T cells, followed by the antiflag IP assay. Samples for co-IP (input) and collection from co-IP were subjected to western blot. FL: full-length; *Δ*AF2: deletion from AF2 (activation function 2 domain) to the C-terminal; *Δ*LBD: deletion from LBD (ligand-binding domain) to the C-terminal; *Δ*DBD: deletion from DBD (DNA-binding domain) to the C-terminal; AF1: activation function 1 domain.

Because protein-protein interaction is involved in cascade events of UPS-mediated protein degradation, the yeast two-hybrid (Y2H) system was applied to screen NR4A3-binding proteins. Due to the autoactivation effect of the bait vector with the encoding fragment of N-terminal 1-292-amino acids of NR4A3 in yeast (data not shown), the DNA fragment encoding C-terminal 308 amino acids (320-627 aa) of NR4A3 (NR4A3-C) was cloned into the plasmid pGB and served as a bait for Y2H screening. Two positive candidate colonies were isolated and sequenced, and the DNA fragments harbored in these two colonies shared high identity to cDNA of SMARCB1 and lymphocyte cell-specific protein-tyrosine kinase (LCK) by BLASTn search, respectively (Figure S2). PCR products encoding NR4A3-C and full-length cDNA of SMARCB1 or LCK were constructed into the mammalian expression plasmids pCDEF-flag and pCDEF-HA, respectively, and then, two plasmids were cotransfected into HEK-293T cells. Then, coimmunoprecipitation (co-IP) was conducted to verify the interactions. As shown in Figure 4(a), SMARCB1 was found to interact with NR4A3-C in the co-IP assay, while the interaction between LCK and NR4A3-C was not detected (data not shown). To map the minimal binding region of NR4A3, five plasmids expressing full-length and four truncated NR4A3 with different domain deletions were constructed (Figure 4(b)) and were coexpressed with SMARCB1 in HEK-293T cells, respectively. The co-IP results showed that SMARCB1 did not bind with NR4A3 truncations which were lacking the DBD domain (Figure 4(c)), indicating that the DBD domain of NR4A3 is required for NR4A3-SMARCB1 interaction.

### 3.5. SMARCB1 Mediates the Ubiquitination of NR4A3

**Figure 5:**
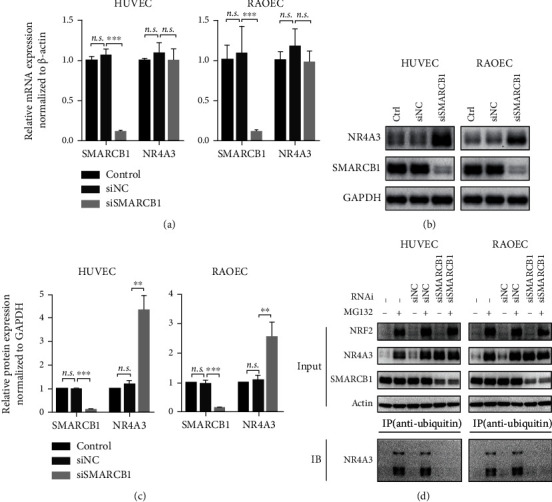
SMARCB1 mediates the ubiquitination of NR4A3. (a) Relative mRNA expression of NR4A3 in HUVEC (left panel) and RAOEC (right panel) while SMARCB1 being knockdown. (b) Representative blot of protein expression of NR4A3 in HUVEC (left panel) and RAOEC (right panel) while SMARCB1 being knockdown. (c) Quantification of NR4A3 protein expression was determined by the grayscale of blots from three independent experiments and normalized to GAPDH. (d) Determination of ubiquitination of NR4A3 in HUVEC (left panel) and RAOEC (right panel) while SMARCB1 being knockdown by the antiubiquitin IP assay followed by western blot. After SMARCB1 being knockdown for 24 hours, 100 nM MG132 was applied for an additional 24-hour incubation followed by sample collection for the IP assay. Statistical analysis of (a) and (c) was performed using one-way ANOVA, followed by Tukey's multiple comparison tests; *n.s.*: no significance; ^∗∗^*p* < 0.01 and ^∗∗∗^*p* < 0.001. RNAi: RNA interference; Ctrl: normal cultured cells; siNC: cells subjected to treatment with negative scrambled small interfering RNA; siSMARCB1: cells subjected to treatment with small interfering RNA against SMARCB1.

A single research reported the possibility that SMARCB1 might be involved in regulating UPS activity through modulating the transcription of UPS-related proteins [35]. Moreover, using computational modeling, the conserved imperfect repeat unit (Rpt1) domain of SMARCB1 was identified to share structural similarity with the PLAA family of the ubiquitin-binding (PFU) domain of phospholipase A2-activating protein (PLAA) which is required for the interaction with ubiquitin [36]. PLAA, a mammalian homolog of yeast ubiquitin fusion degradation protein 3 (UFD3) [37], is proposed to play a role in UPS-mediated degradation of ubiquitylated proteins through its association with valosin-containing protein (VCP) [38–40]. These reports suggested that SMARCB1 may participate in the process of protein ubiquitylation or UPS-mediated protein degradation. However, whether SMARCB1 can directly mediate NR4A3 ubiquitination in VEC is unknown. We firstly knocked down SMARCB1 in HUVEC and RAOEC in normal culture condition and found that the reduction of SMARCB1 did not affect the NR4A3 mRNA level (Figure 5(a)) but led to a significant evaluation of the NR4A3 protein content (Figures 5(b) and 5(c)). Further, the treatment with the proteasome inhibitor MG132 increased the ubiquitination of NR4A3, which was abolished by knockdown of SMARCB1 (Figure 5(d)), supporting the idea that SMARCB1 is a regulator for NR4A3 ubiquitination. Besides, knockdown of SMARCB1 did not result in NF-E2-related factor 2 (NRF2) accumulation which is a well-known protein degraded by UPS (Figure 5(d)), suggesting that knockdown of SMARCB1 did not impair the UPS activity and further supported the idea that SMARCB1 specifically regulated ubiquitination of NR4A3. In conclusion, our results support the idea that SMARCB1 promotes the ubiquitination of NR4A3 by direct interaction.

### 3.6. SMARCB1-NR4A3 Interaction Is Enhanced by ROS

**Figure 6:**
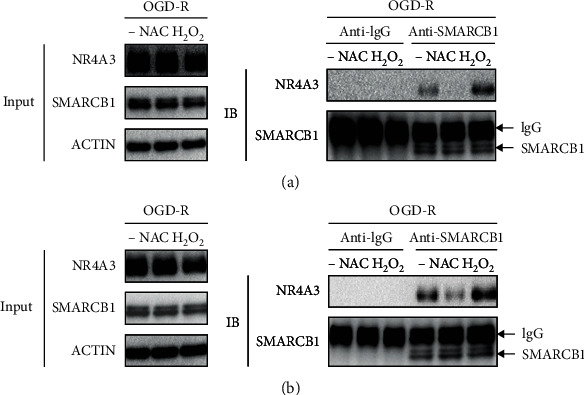
SMARCB1-NR4A3 interaction is enhanced by ROS. Determination of the effect of NAC or H_2_O_2_ on SMARCB1-NR4A3 interaction in (a) HUVEC or in (b) RAOEC under reoxygenation condition by the anti-SMARCB1 IP assay followed by western blot. Cells were subjected to 4-hour OGD treatment and reoxygenation for 1 hour (OGD-R). 4 mM NAC and 500 *μ*M H_2_O_2_ were applied at the beginning of reoxygenation. For the co-IP assay, an isotype IgG was used to serve as a negative control.

The above results showed that OGD-R-induced ROS promoted NR4A3 degradation in VEC, and whether ROS enhanced the interaction of SMARCB1 and NR4A3 needed to be verified. HUVEC (Figure 6(a)) or RAOEC (Figure 6(b)) were subjected to 4-hour OGD treatment followed by 1-hour reoxygenation incubated with H_2_O_2_ or antioxidant NAC, respectively, and then, the interaction between SMARCB1 and NR4A3 was evaluated by co-IP. The H_2_O_2_ treatment significantly enhanced the interaction which was greatly abolished by NAC treatment in both HUVEC and RAOEC (Figures 6(a) and 6(b)). Our results strongly supported the idea that ROS promotes NR4A3 degradation by enhancing the SMARCB1-NR4A3 interaction.

**Figure 7:**
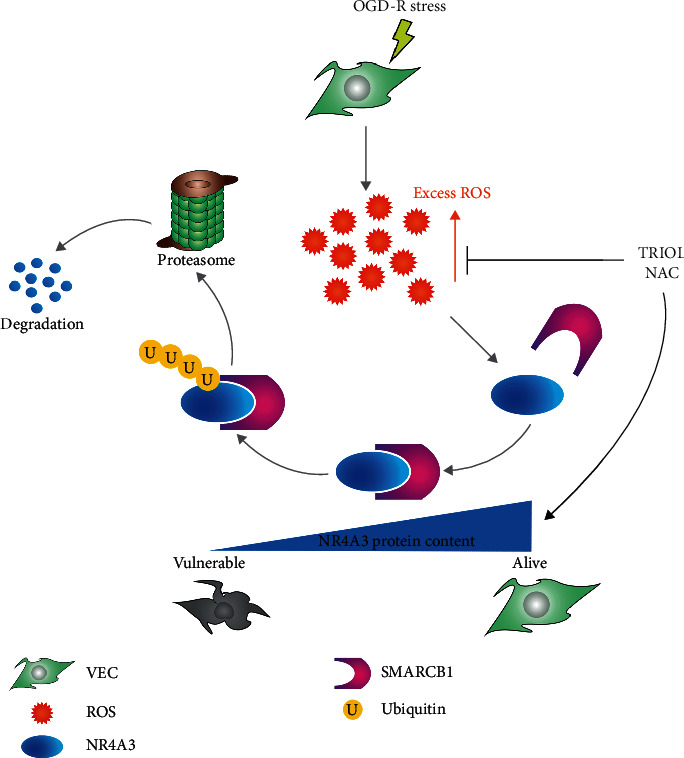
Schematic diagram of the putative working model for SMARCB1-mediated ubiquitination of NR4A3.

In summary, our study demonstrates that excess of ROS promotes the UPS-mediated NR4A3 degradation by enhancing the interaction of NR4A3 and SMARCB1 which was required for the ubiquitination of NR4A3 in VEC under OGD-R condition, and TRIOL or NAC can attenuate the overproduction of ROS and inhibit the NR4A3 degradation (see Figure 7 for the schematic diagram).

## 4. Discussion

The increased NR4A3 plays a protective role in different types of cells [8, 10, 12], suggesting the vital importance of NR4A3 for cell survival regulation against stresses. NR4A3 is generally believed to be transcriptionally regulated [1, 2], and our study here reveals that the ubiquitination and degradation also contribute to modulating the NR4A3 protein content which is driven by ROS accumulation in VEC injury. Moreover, our results indicate that this UPS-mediated NR4A3 degradation may be a universal mechanism in both the physiological and pathological processes, and the NR4A3 level depends on the dynamic balance of regulations between transcription and protein degradation. As shown in Figure 5(d), in the presence of oxygen, NR4A3 ubiquitination is detectable and its degradation could be blocked by the proteasome inhibitor MG132, suggesting that UPS-mediated NR4A3 reduction modulates the NR4A3 protein level in physical condition. Under OGD condition, consistent with previous studies [10, 41], NR4A3 mRNA is dramatically upregulated (Figure 3(a)), supporting the idea that the transcription activation of NR4A3 possibly by HIF1-*α* is a predominant pattern to regulate the NR4A3. Under OGD-R condition, although accompanied by the decreased NR4A3 mRNA level (Figure 3(a)), our results support the idea that ubiquitination and degradation of NR4A3 enhanced by ROS also play a crucial role in NR4A3 reduction since blockade of protein synthesis by CHX results in almost complete exhaustion of NR4A3 protein (Figure 3(b)).

SMARCB1 participates in various biological processes including cell proliferation and differentiation, cellular antiviral activities and inhibition of tumor formation by chromatin remodeling, epigenetic modifications of histone, and even modulation of transcription factor activity via biochemical interactions with different biomolecules [18, 20, 42]. Our study for the first time reveals its function in regulating the protein content via UPS in a manner of posttranslational modification via direct interaction to modulate targets' function such as the ligand-independent transcription factor NR4A3, further expanding the understanding on the regulation of the SMARCB1-mediated biochemical process in VEC. Here, ROS is supposed to be a “catalyzer” for SMARCB1 binding to NR4A3; however, the detailed mechanism needs to be further investigated. The interaction identified in this study together with the structural similarity between the Rpt1 domain of SMARCB1 and PFU domain of PLAA which is identified to be associated with ubiquitin binding [36] suggests that SMARCB1 may act as an E3 ubiquitin ligase which promotes target ubiquitination and UPS-mediated degradation. We also identify the important role of DBD of NR4A3 in the SMARCB1-NR4A3 interaction. Interestingly, NR4A receptors share highly conserved DBD (degree of conservation > 90%) [11, 43, 44], suggesting that SMARCB1 may also regulate other members of the NR4A receptor family including NR4A1 and NR4A2. Our study not only identifies a novel posttranslational modulation of NR4A3 in VEC injury but also provides new clues related to SMARCB1 to explore the regulatory mechanism of pathophysiological processes involving NR4A receptors including metabolic disease, inflammation, cardiovascular disease, neurological functions, immune responses, and cancer.

Our study here verifies the protective role of the TRIOL- and NR4A3-related mechanism in VEC and the pulmonary endothelium against OGD-R/hypoxia injury, supporting the idea that TRIOL serves as a candidate agent for the treatment of ALI. Previous studies on the protective mechanism of TRIOL reveal the “nongenomic effects” of negative modulation on intracellular Ca^2+^ overload induced by glutamate in neurons and the “genomic effects” of transcriptional profile change in brain tissue and white blood cells [32]. Furthermore, TRIOL not only activates the antioxidative NRF2 pathway [30] but also upregulates NR4A3, both of which contribute to the “genomic effects” of TRIOL. What is more, TRIOL sufficiently inhibits the excess production of ROS in VEC under OGD-R condition and in neuron [29] while ROS notably promotes NR4A3 protein degradation through enhancing the interaction of NR4A3 and SMARCB1. Since ROS overproduction is well documented in various pathological injuries including vascular endothelium impairment under high glucose or inflammation conditions [45–48], it is worthy to further explore the feasibility of TRIOL on treating endothelial barrier impairment in diabetes or sepsis.

## 5. Conclusions

In summary, our study reveals the ROS-driven ubiquitination and degradation of NR4A3 under OGD-R condition and identifies that SMARCB1 is an important regulator for the modification of NR4A3 ubiquitination by direct interaction and provides the promising candidate antioxidant TRIOL for treating pulmonary endothelial barrier impairment in ALI.

## Data Availability

The data used to support the findings of this study are included within this article and the supplementary information files of this article.

## Supplementary Materials

Supplementary Materials Figure S1: RPKM of NR4A receptors in the brain tissue of cynomolgus monkeys. Figure S2: nucleic acid sequences of positive clones identified by yeast two-hybrid screening.
