# Supplementary material for: SMARCB1 Promotes Ubiquitination and Degradation of NR4A3 via Direct Interaction Driven by ROS in Vascular Endothelial Cell Injury
Source: Oxid Med Cell Longev. 2020 Oct 23;2020:2048210. doi: 10.1155/2020/2048210 (PMC7604603; doi:10.1155/2020/2048210)
Supplement: Supplementary Materials — Figure S1: RPKM of NR4A receptors in the brain tissue of cynomolgus monkeys. Figure S2: nucleic acid sequences of positive clones identified by yeast two-hybrid screening. [file 2048210.f1.docx]

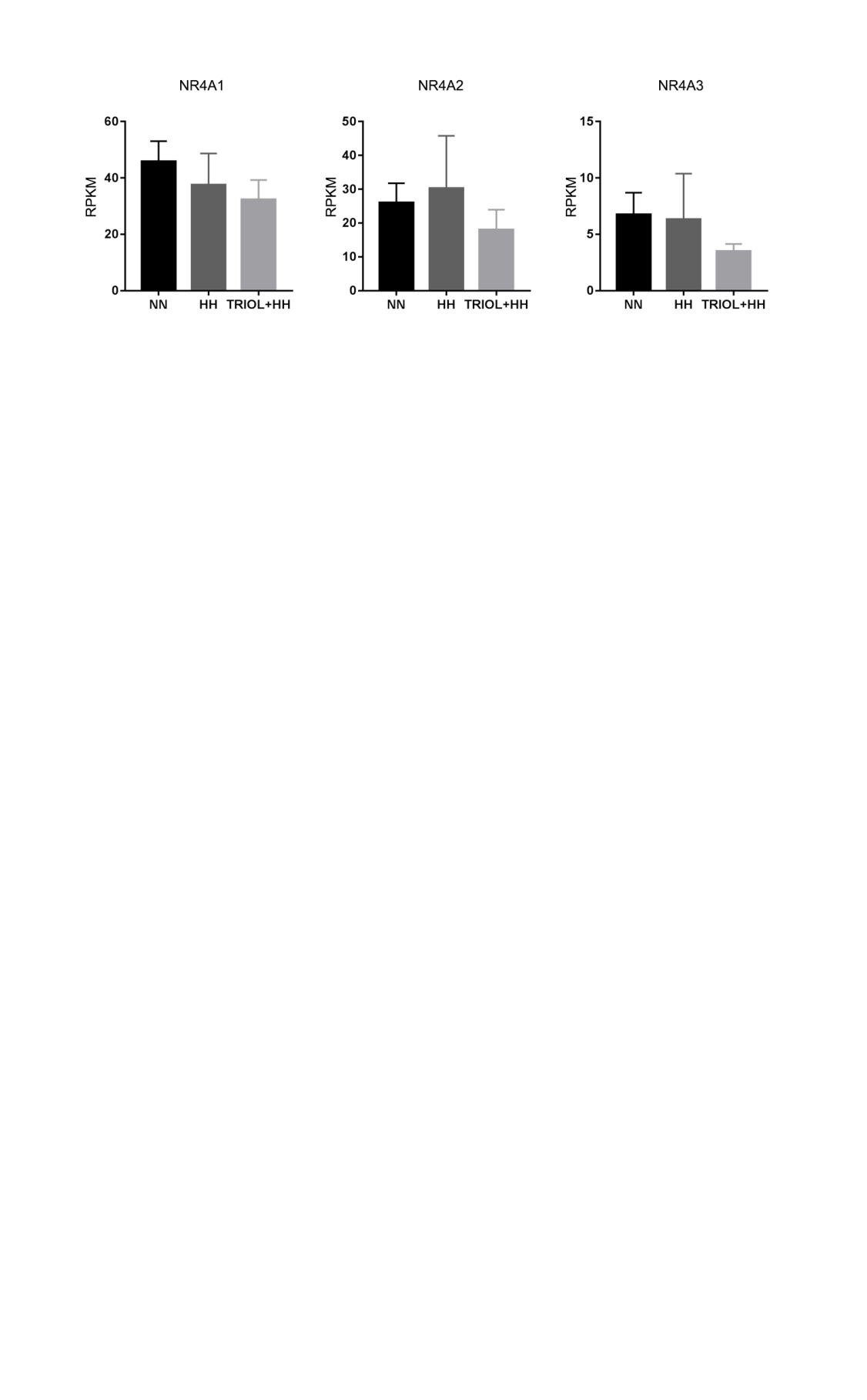


**Fig s1: RPKM of NR4As receptors in brain tissue of cynomolgus monkeys.** RPKM, reads per kilo base per million mapped reads. No significant difference was observed in comparisons of RPKM of each NR4A receptors between NN, HH, and TRIOL+HH groups. The original sequencing data was deposited with the National Center for Biotechnology Information (NCBI) under accession No. PRJNA431946 and in the CNSA (https://db.cngb.org/cnsa/) of CNGBdb with accession code CNP0000293 [32]. In this study, the comparisons of RPKM of each NR4A receptors were reorganized. n = 6~7/group.


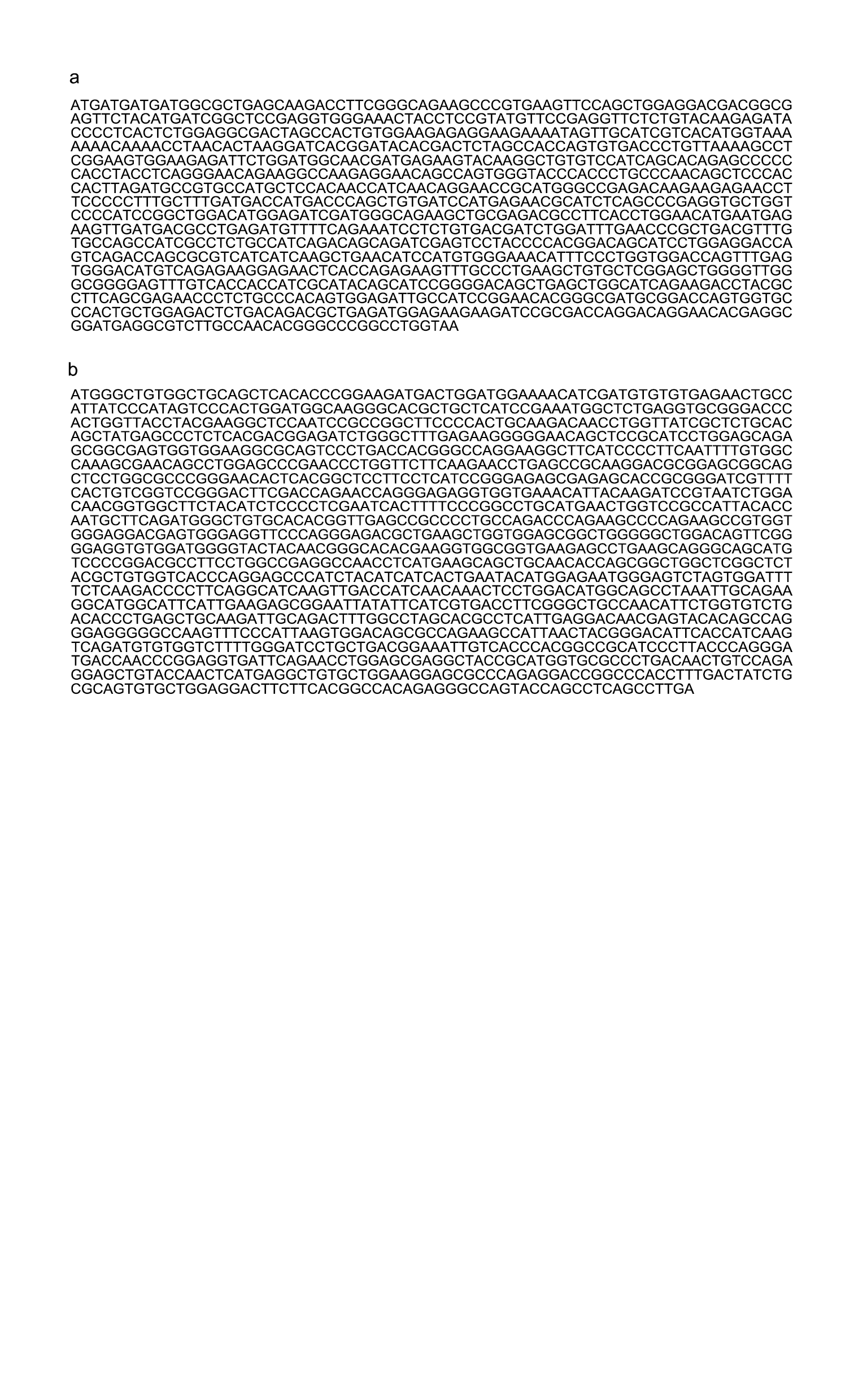


**Fig s2: Nucleic acid sequences of positive clones identified by yeast two hybrid screening.** (a) Sequence of positive clone 1 shows 99.74% identity to human SMARCB1 (NP_003064.2), 99.74% to mouse SMARCB1 (NP_035548.1) and 97.40% to rat SMARCB1 (NP_001020899.1), respectively. (b) Sequence of positive clone 2 that shows 99.61% identity to human LCK (NP_001036236.1), 96.46% to mouse LCK (NP_001155904.1) and 96.46% to rat LCK (XP_032752966.1), respectively. Sequence identification was performed by BLASTn at the website of National Center for Biotechnology Information. (<https://blast.ncbi.nlm.nih.gov>).

The sequences in Fig s2 are provided as follow.

a

ATGATGATGATGGCGCTGAGCAAGACCTTCGGGCAGAAGCCCGTGAAGTTCCAGCTGGAGGACGACGGCGAGTTCTACATGATCGGCTCCGAGGTGGGAAACTACCTCCGTATGTTCCGAGGTTCTCTGTACAAGAGATACCCCTCACTCTGGAGGCGACTAGCCACTGTGGAAGAGAGGAAGAAAATAGTTGCATCGTCACATGGTAAAAAAACAAAACCTAACACTAAGGATCACGGATACACGACTCTAGCCACCAGTGTGACCCTGTTAAAAGCCTCGGAAGTGGAAGAGATTCTGGATGGCAACGATGAGAAGTACAAGGCTGTGTCCATCAGCACAGAGCCCCCCACCTACCTCAGGGAACAGAAGGCCAAGAGGAACAGCCAGTGGGTACCCACCCTGCCCAACAGCTCCCACCACTTAGATGCCGTGCCATGCTCCACAACCATCAACAGGAACCGCATGGGCCGAGACAAGAAGAGAACCTTCCCCCTTTGCTTTGATGACCATGACCCAGCTGTGATCCATGAGAACGCATCTCAGCCCGAGGTGCTGGTCCCCATCCGGCTGGACATGGAGATCGATGGGCAGAAGCTGCGAGACGCCTTCACCTGGAACATGAATGAGAAGTTGATGACGCCTGAGATGTTTTCAGAAATCCTCTGTGACGATCTGGATTTGAACCCGCTGACGTTTGTGCCAGCCATCGCCTCTGCCATCAGACAGCAGATCGAGTCCTACCCCACGGACAGCATCCTGGAGGACCAGTCAGACCAGCGCGTCATCATCAAGCTGAACATCCATGTGGGAAACATTTCCCTGGTGGACCAGTTTGAGTGGGACATGTCAGAGAAGGAGAACTCACCAGAGAAGTTTGCCCTGAAGCTGTGCTCGGAGCTGGGGTTGGGCGGGGAGTTTGTCACCACCATCGCATACAGCATCCGGGGACAGCTGAGCTGGCATCAGAAGACCTACGCCTTCAGCGAGAACCCTCTGCCCACAGTGGAGATTGCCATCCGGAACACGGGCGATGCGGACCAGTGGTGCCCACTGCTGGAGACTCTGACAGACGCTGAGATGGAGAAGAAGATCCGCGACCAGGACAGGAACACGAGGCGGATGAGGCGTCTTGCCAACACGGGCCCGGCCTGGTAA

b

ATGGGCTGTGGCTGCAGCTCACACCCGGAAGATGACTGGATGGAAAACATCGATGTGTGTGAGAACTGCCATTATCCCATAGTCCCACTGGATGGCAAGGGCACGCTGCTCATCCGAAATGGCTCTGAGGTGCGGGACCCACTGGTTACCTACGAAGGCTCCAATCCGCCGGCTTCCCCACTGCAAGACAACCTGGTTATCGCTCTGCACAGCTATGAGCCCTCTCACGACGGAGATCTGGGCTTTGAGAAGGGGGAACAGCTCCGCATCCTGGAGCAGAGCGGCGAGTGGTGGAAGGCGCAGTCCCTGACCACGGGCCAGGAAGGCTTCATCCCCTTCAATTTTGTGGCCAAAGCGAACAGCCTGGAGCCCGAACCCTGGTTCTTCAAGAACCTGAGCCGCAAGGACGCGGAGCGGCAGCTCCTGGCGCCCGGGAACACTCACGGCTCCTTCCTCATCCGGGAGAGCGAGAGCACCGCGGGATCGTTTTCACTGTCGGTCCGGGACTTCGACCAGAACCAGGGAGAGGTGGTGAAACATTACAAGATCCGTAATCTGGACAACGGTGGCTTCTACATCTCCCCTCGAATCACTTTTCCCGGCCTGCATGAACTGGTCCGCCATTACACCAATGCTTCAGATGGGCTGTGCACACGGTTGAGCCGCCCCTGCCAGACCCAGAAGCCCCAGAAGCCGTGGTGGGAGGACGAGTGGGAGGTTCCCAGGGAGACGCTGAAGCTGGTGGAGCGGCTGGGGGCTGGACAGTTCGGGGAGGTGTGGATGGGGTACTACAACGGGCACACGAAGGTGGCGGTGAAGAGCCTGAAGCAGGGCAGCATGTCCCCGGACGCCTTCCTGGCCGAGGCCAACCTCATGAAGCAGCTGCAACACCAGCGGCTGGCTCGGCTCTACGCTGTGGTCACCCAGGAGCCCATCTACATCATCACTGAATACATGGAGAATGGGAGTCTAGTGGATTTTCTCAAGACCCCTTCAGGCATCAAGTTGACCATCAACAAACTCCTGGACATGGCAGCCTAAATTGCAGAAGGCATGGCATTCATTGAAGAGCGGAATTATATTCATCGTGACCTTCGGGCTGCCAACATTCTGGTGTCTGACACCCTGAGCTGCAAGATTGCAGACTTTGGCCTAGCACGCCTCATTGAGGACAACGAGTACACAGCCAGGGAGGGGGCCAAGTTTCCCATTAAGTGGACAGCGCCAGAAGCCATTAACTACGGGACATTCACCATCAAGTCAGATGTGTGGTCTTTTGGGATCCTGCTGACGGAAATTGTCACCCACGGCCGCATCCCTTACCCAGGGATGACCAACCCGGAGGTGATTCAGAACCTGGAGCGAGGCTACCGCATGGTGCGCCCTGACAACTGTCCAGAGGAGCTGTACCAACTCATGAGGCTGTGCTGGAAGGAGCGCCCAGAGGACCGGCCCACCTTTGACTATCTGCGCAGTGTGCTGGAGGACTTCTTCACGGCCACAGAGGGCCAGTACCAGCCTCAGCCTTGA
